# Antiplatelet Strategies for Older Patients with Acute Coronary Syndromes: Finding Directions in a Low-Evidence Field

**DOI:** 10.3390/jcm12052082

**Published:** 2023-03-06

**Authors:** Stefano De Servi, Antonio Landi, Stefano Savonitto, Nuccia Morici, Leonardo De Luca, Claudio Montalto, Gabriele Crimi, Roberta De Rosa, Giuseppe De Luca

**Affiliations:** 1 Department of Molecular Medicine, University of Pavia Medical School, 27100 Pavia, Italy; 2 Division of Cardiology, Cardiocentro Ticino Institute, Ente Ospedaliero Cantonale (EOC), 6900 Lugano, Switzerland; 3 Clinica San Martino, 23864 Malgrate, Italy; 4 IRCCS S. Maria Nascente—Fondazione Don Carlo Gnocchi ONLUS, 20148 Milan, Italy; 5 Department of Cardiovascular Sciences, A.O. San Camillo-Forlanini, 00152 Roma, Italy; 6 Interventional Cardiology, De Gasperis Cardio Center, Niguarda Hospital, 20162 Milan, Italy; 7 Clinical and Interventional Cardiology, Istituto Clinico Sant’Ambrogio, Gruppo San Donato, 20122 Milan, Italy; 8 Interventional Cardiology Unit, Cardio-Thoraco Vascular Department (DICATOV), IRCCS, Ospedale Policlinico San Martino, 16132 Genova, Italy; 9 University Hospital San Giovanni di Dio e Ruggi d’Aragona, 84131 Salerno, Italy; 10 Goethe University Hospital Frankfurt, 60528 Frankfurt am Main, Germany; 11 Division of Cardiology, AOU “Policlinico G. Martino”, Department of Clinical and Experimental Medicine, University of Messina, 98039 Messina, Italy; 12 Division of Cardiology, Nuovo Galeazzi-Sant’Ambrogio Hospital, 20161 Milan, Italy

**Keywords:** elderly patients, acute coronary syndrome, anti-platelet therapy, antithrombotic therapy, percutaneous coronary intervention, high bleeding risk

## Abstract

Patients ≥ 75 years of age account for about one third of hospitalizations for acute coronary syndromes (ACS). Since the latest European Society of Cardiology guidelines recommend that older ACS patients use the same diagnostic and interventional strategies used by the younger ones, most elderly patients are currently treated invasively. Therefore, an appropriate dual antiplatelet therapy (DAPT) is indicated as part of the secondary prevention strategy to be implemented in such patients. The choice of the composition and duration of DAPT should be tailored on an individual basis, after careful assessment of the thrombotic and bleeding risk of each patient. Advanced age is a main risk factor for bleeding. Recent data show that in patients of high bleeding risk short DAPT (1 to 3 months) is associated with decreased bleeding complications and similar thrombotic events, as compared to standard 12-month DAPT. Clopidogrel seems the preferable P2Y12 inhibitor, due to a better safety profile than ticagrelor. When the bleeding risk is associated with a high thrombotic risk (a circumstance present in about two thirds of older ACS patients) it is important to tailor the treatment by taking into account the fact that the thrombotic risk is high during the first months after the index event and then wanes gradually over time, whereas the bleeding risk remains constant. Under these circumstances, a de-escalation strategy seems reasonable, starting with DAPT that includes aspirin and low-dose prasugrel (a more potent and reliable P2Y12 inhibitor than clopidogrel) then switching after 2–3 months to DAPT with aspirin and clopidogrel for up to 12 months.

## 1. Introduction

Despite the improvements in revascularization techniques [1,2], antithrombotic therapies [3,4,5] and other measures of secondary prevention including lifestyle modifications and pharmacological treatments, coronary artery disease still represents the leading cause of mortality in developed countries [6] and several efforts have been made to identify new risk factors [7,8,9,10] in order to promote and improve primary and secondary prevention. Patients ≥ 75 years of age account for about one-third of hospitalizations of patients with acute coronary syndromes (ACS) [11]. However, these data consider only older patients admitted to Coronary Care Units or Cardiology wards and underestimate the true number of those hospitalized for ACS. In a prospective, multicentre study using principles of clinical governance [12], aiming to verify and quantify consecutive inclusion of hospitalized ACS patients, it was found that only 69.5% of patients admitted with a diagnosis of ACS were included, due to the prevalent enrolment by participating centres of patients admitted to cardiac wards. Since the greater number of ACS patients admitted to non-cardiac wards are older patients with multiple comorbidities and/or geriatric syndromes, these data show that current epidemiological data underestimate the true incidence of patients with advanced age in ACS series. Moreover, the mortality rates are greatly affected by the characteristics of the population samples included in randomized trials or in observational registries. In the elderly-ACS trial of non-ST-elevation ACS [13], the 1-year mortality rate in randomized patients was 13%, whereas it was 23% in those included in the concurrent registry and not randomized [14]. Patients enrolled in randomized trials, even in those designed for elderly patients, are the fittest ones and do not reflect the wide spectrum of clinical conditions associated with advanced age. It is likely that mortality rates are even higher than those reported in observational studies, due to the exclusion of “neglected” older patients admitted with ACS in medical wards.

Age itself does not accurately mirror the patient’s status, as other features such as comorbidities and geriatric syndromes (frailty, disability, cognitive impairment) are the factors determining patient health and outcomes [15,16,17,18]. Frailty represents a clinical condition associated with increased vulnerability to endogenous or exogenous stressors [16]. It is present in 25–50% of older adults >85 years admitted with ACS, although these figures depend on the definition applied [17]. Cognitive impairment is frequently found in frail, older patients with myocardial infarction (MI) [18], a condition frequently associated with the presence of diabetes, smoking, and the metabolic syndrome, all established risk factors for coronary artery disease and worse long-term outcomes [19,20]. Moreover, these patients are more likely to have major adverse cardiovascular events at follow-up, leading to further cognitive decline [21]. In presence of such limited evidence and knowledge gaps it is understandable that current guidelines do not give straightforward recommendations for these patients [22], simply indicating that the care for older adults with ACS should be carefully provided, weighing on an individual basis the risk versus benefit for the patient not only when initially choosing an invasive versus a conservative strategy, but also when considering pharmacological therapies, particularly antithrombotic drugs for secondary prevention. In the present article, we reviewed all available evidence on antithrombotic treatments in older ACS patients, including novel treatment options based on the individual bleeding and ischemic risk, such as short dual antiplatelet therapy and de-scalation strategies.

## 2. Invasive versus Conservative Strategy

Although the European Society of Cardiology (ESC) STEMI guidelines state that “there is no upper age limit with respect to reperfusion, especially with primary PCI” [23], there is relatively little information regarding the outcomes of elderly patients undergoing primary PCI, due to the low representation of elderly patients in clinical trials assessing the effects of mechanical reperfusion for STEMI. A pooled analysis [24] including 834 patients enrolled in three randomized trials (Zwolle [25], SENIOR PAMI [26], and TRIANA [24]) showed that the overall risk of death, re-infarction, or disabling stroke was substantially lower for patients allocated to primary PCI compared with those treated with fibrinolysis (14.9% vs. 21.5%; odds ratio [OR], 0.64; 95% confidence interval [CI] 0.45–0.91; *p* = 0.013), and s only a trend toward reduction of death was found (10.7% versus 13.8%, hazard ratio [HR] 0.74, 95% CI 0.49–1.13), although the effect size was superimposable to that of the largest metanalysis comparing fibrinolysis and primary PCI in younger patients. Septuagenarians and octogenarians undergoing primary PCI show higher mortality rates, both at short-term and mid-term follow-up than younger patients. Registry data [11,27] indicate a growing number of primary PCI procedures in the older-patient population with STEMI, accompanied by a progressive reduction in early mortality. Despite these improvements, a recent analysis of two centres, including 3.411 STEMI patients treated with primary PCI, showed that both early and late mortality rates progressively increase as age advances: at 1-month, 19% of octogenarians, 12.3% of septuagenarians and 2.9% of younger patients died (*p* = 0.01), whereas the respective mortality rates at 3 years were 27.4%, 19.3% and 4.7% (*p* < 0.01). On the contrary, rates of major adverse-cardiovascular events as well as target-vessel revascularization and stent thrombosis were similar between the two groups, both at 1 month and at 3 years [28].

Although more data are available from randomized trials conducted in elderly patients with non-ST segment elevation ACS (NSTE-ACS) than in STEMI, the impact of these results on clinical practice is lower than for STEMI patients. The different clinical presentation on admission (patients with STEMI have ongoing ischemia, whereas most patients with NSTEACS are asymptomatic), cautions against an immediate invasive treatment and encourages one to be initially conservative, despite the evidence from randomized trials being in favour of an interventional approach. A benefit from an interventional approach was observed in the post hoc analysis of the older patients included in the TACTIS-TIMI 18 (Treat Angina with Aggrastat and Determine Cost of Therapy with an Invasive or Conservative Strategy—Thrombolysis in Myocardial Infarction 18) trial [29] and in an individual-patient data analysis (FIR collaboration) of the FRISC II (Fast Revascularization during Instability in Coronary artery disease), ICTUS (Invasive vs. Conservative Treatment in Unstable Coronary Syndromes), and RITA-3 (Randomized Intervention Trial of unstable Angina Investigators) trials [30]: in patients 75 years old or older, the routine invasive strategy was associated with a lower risk of cumulative adverse events (unadjusted HR 0.71, 95% CI 0.55–0.91, *p* = 0.007), whereas no benefit was observed in patients <65 years (HR 1.11, 95% CI 0.90–1.38, *p* = 0.33). 

In dedicated randomized trials in NSTE-ACS patients, the Italian Elderly ACS trial [13,31], which enrolled 313 patients with NSTE-ACS aged ≥75 years; the After Eighty trial [32], which randomized 557 patients with NSTE-ACS aged ≥80 years; and the RINCAL trial [33] that included 251 patients (Table 1), the results went in the same direction: older patients allocated to the routine invasive strategy had a lower risk of death and MI, as shown by a meta-analysis (OR 0.65, 95% CI 0.51–0.83; *p* < 0.001) at a median follow-up of 36 months. This result was mostly driven by a statistically significant reduction in MI with a trend towards a lower mortality rate, without heterogeneity among the studies [34]. A significant reduction in mortality was, however, found in the observational SENIOR NSTEMI cohort study that included patients aged >80 years: applying a propensity-score model, this study showed that at 5 years the adjusted risk of dying was 44% lower with early invasive treatment, with the difference emerging from 1 year onwards [35]. The ongoing SENIOR-RITA trial is randomizing a large series of NSTEMI patients aged ≥75 years to determine the impact of a routine invasive strategy on cardiovascular death and non-fatal MI, compared with a conservative treatment strategy [36].

The 2021 European Society of Cardiology guidelines on NSTE-ACS recommend that older patients use the same diagnostic and interventional strategies used for the younger ones [22]. However, since patients included in randomized clinical trials are the fittest ones in their age category [15,18], the guidelines exhort considering the risk–benefit trade-off of an invasive approach, estimated life expectancy, comorbidities, quality of life, frailty, cognitive and functional impairment. Frail patients are less likely to receive coronary angiography and PCI, due to the perception, not based on clinical evidence, of the risk associated with revascularization procedures in these patients [37]. In the Spanish LONGEVO registry, non-frail octogenarians with ACS treated conservatively showed a higher rate of cardiac death, reinfarction, or new revascularization at six months, whereas frail patients did not show any apparent benefit from an invasive approach [38]. Other observational data, however, suggest a better outcome in frail patients with NSTE-ACS when treated with PCI [39,40]. More data are needed in this setting before definite conclusions are reached.

## 3. Dual Antiplatelet Therapy in Elderly ACS Patients: Comparative Efficacy and Safety among Different P2Y_12_ Inhibitors

Data on optimal platelet inhibition in older adults is limited [41], because elderly patients were underrepresented in the pivotal trials: they accounted for only 13% of patients in the TRITON-TIMI 38 trial (Trial to Assess Improvement in Therapeutic Outcomes by Optimizing Platelet Inhibition with Prasugrel—Thrombolysis in Myocardial Infarction study) [42] and for 15% in the PLATO (The Study of Platelet Inhibition and Patient Outcomes) trial [43]. Dual antiplatelet therapy (DAPT) with prasugrel at 10 mg daily dose associated with aspirin significantly increased bleeding in the TRITON-TIMI 38 trial as compared to DAPT with clopidogrel [42], so that its use in elderly patients was not recommended by the Food and Drug Administration, whereas the European Medicines Agency indicated a 5 mg/day maintenance dose [44]. On the contrary, an analysis of the PLATO trial showed that the superiority of DAPT with ticagrelor over DAPT with clopidogrel (including a reduction in cardiovascular mortality) was confirmed also in the elderly population [45]. These indications were issued despite the fact that the differences in the primary endpoint of death, MI and stroke between clopidogrel and prasugrel in the TRITON-TIMI 38 trial (18.3% vs. 17.2%) [42] and those between clopidogrel and ticagrelor in the PLATO trial (18.3% vs. 17.2%) [45] were exactly the same. Moreover, a sub-analysis of the PLATO trial on patients undergoing revascularization during the index admission, and therefore comparable to the TRITON-TIMI 38 trial population, found a benefit for ticagrelor over clopidogrel in patients <65 years of age (OR 0.59, CI 0.41–0.85), but not in those aged ≥65 years (OR 1.17, CI 0.85–1.61; interaction *p* < 0.01). 

In clinical practice, the choice of antiplatelet agents in older ACS patients is difficult, since these patients are more prone to bleeding than younger ones, due to the presence of clinical comorbidities that increase bleeding risk and may impact on mortality [46,47,48]. 

Specific trials have been conducted in older ACS patients, comparing different P2Y12 inhibitors in association with aspirin (Table 2). The ELDERLY ACS 2 trial randomized 1443 ACS patients aged ≥75 years who underwent PCI and showed similar combined thrombotic and bleeding events in patients assigned to 12-month DAPT with a prasugrel 5 mg maintenance dose and in those assigned to 12-month DAPT with clopidogrel 75mg [49]. In a post hoc analysis, DAPT with prasugrel 5 mg, as compared to DAPT with clopidogrel, reduced thrombotic events in the first month after the index event, but increased late bleeding (31–365 days) [50]. DAPT with low-dose prasugrel and clopidogrel also had similar efficacy and safety in medically treated elderly patients enrolled in the TRILOGY ACS (Targeted Platelet Inhibition to Clarify the Optimal Strategy to Medically Manage Acute Coronary Syndromes) study [51]. Furthermore, no benefit was found in the ANTARCTIC (Assessment of a Normal vs. Tailored Dose of Prasugrel After Stenting in Patients Aged >75 Years to Reduce the Composite of Bleeding, Stent Thrombosis and Ischaemic Complications) trial by adjusting the dose of prasugrel after 2–4 weeks, based on the results of platelet-function testing [52].

At odds with the results of the post hoc analysis of the PLATO trial, the POPular AGE (Ticagrelor or Prasugrel Versus Clopidogrel in Elderly Patients With an Acute Coronary Syndrome and a High Bleeding Risk: Optimization of Antiplatelet Treatment in High-Risk Elderly) trial showed that DAPT with clopidogrel had significantly lower bleeding rates (including fatal bleeding) compared with DAPT with ticagrelor (17.6% vs. 23.1%; OR 0.74; 95% CI 0.56–0.97), without any difference in thrombotic events (12.8% vs. 12.5%; OR 1.02, 95% CI 0.72–1.45) [53]. Notably, ticagrelor was prematurely discontinued in about half of the patients randomly allocated to that drug, a finding that could have hampered its potential benefits, but that also indicates that side effects induced by that drug affect a large part of older adults. Similar data were found in the SWEDEHEART (Swedish Web System for Enhancement and Development of Evidence-Based Care in Heart Disease Evaluated According to Recommended Therapies) registry, which included 14,005 patients aged ≥80 years discharged on aspirin associated with either clopidogrel (60.2%) or ticagrelor (39.8%) after MI [54]: after statistical adjustment, patients on ticagrelor had a significantly higher risk of death and bleeding compared with those taking clopidogrel. A dedicated analysis of the Praise registry showed comparable results between the two drugs [55]. 

Interestingly, in both the ELDERLY-ACS 2 and POPular AGE trials, thrombotic and bleeding-event rates at 1 year in patients randomized to clopidogrel were far lower than in older patients randomized to clopidogrel in the TRITON-TIMI 38 and PLATO trials [56]. Although clopidogrel has a large response variability, resulting in a non-negligible proportion of patients with high on-treatment platelet reactivity [57], improvement in stent technology [58,59] and increased operator expertise may have hindered the antithrombotic advantage provided by ticagrelor and prasugrel over clopidogrel observed in the first pivotal studies comparing P2Y12 inhibitors with an antiplatelet action of different intensity.

## 4. Bleeding and Thrombotic Risk in Elderly ACS Patients

The goal of the antiplatelet therapy after ACS is to reduce the risk of recurrence of ischemic events, likewise attenuating the bleeding risk [60]. The choice of the composition and optimal duration of DAPT [61,62] should be made on an individual basis, and its effects repeatedly verified throughout the follow-up period. Therefore, cardiologists should assess the thrombotic and bleeding risk of each patient by considering clinical, anatomical, procedural and laboratory data. To this purpose, risk scores, especially for the measurement of the bleeding risk such as the PRECISE DAPT score [63] and the Academic Research Consortium High Bleeding Risk (ARC-HBR) criteria [64,65], may be helpful, and are recommended by guidelines [66]. 

Advanced age is a main risk factor for bleeding. It is included in the PRECISE DAPT score that consists of five variables (age, haemoglobin, creatinine clearance, white blood cell count, history of bleeding) and was developed to predict a 12-month bleeding risk, selecting patients suitable for a short DAPT strategy (those with a score value =>25) [63,66]. Older age is an important determinant of the score: consider a patient 80 years old without anaemia (haemoglobin 13 g/dL) no bleeding history, with a creatinine clearance of 60 mL/min and normal white blood cell count (7000 × 10^9^/L). His calculated score is 26, which denotes a high bleeding risk. Moreover, almost all elderly patients admitted for ACS exceed the proposed cut-off for HBR of the PRECISE DAPT score, due to the very frequent concomitant presence of variables also related to bleeding [67]. 

The ARC-HBR criteria list biochemical and clinical data, and are ranked as major and minor conforming to whether the expected annual bleeding risk is ≥4% or <4%, respectively [59]. Patients with HBR are those with at least one major, or two minor, criteria. Age =>75 years is considered a minor HBR criterion, and thus patients of that age need an additional minor criterion to be defined as HBR. However, recent validation studies [68,69] found that advanced age conveys a major bleeding risk, exceeding 4% (the threshold established for the definition of major HBR criteria), with the risk of bleeding rising in parallel with age [70]. 

Although almost all elderly patients satisfy the criteria for the definition of HBR, high thrombotic risk is also concomitant in many patients. This issue is well outlined in the ARC-HBR trade-off model proposed by Urban et al., who reported the results of 1-year clinical outcome of 6641 patients (26% with STEMI or NSTEMI) who underwent PCI with stent implantation and were categorized as HBR according to ARC criteria [71]. Prior MI, the presence of diabetes, STEMI presentation and bare-metal-stent implantation were predictors of MI and stent thrombosis in this HBR population. At the 1-year follow-up, slightly less than half of the patients (44.1%) had a greater risk of thrombotic events than major bleeding, and one third of patients faced a comparable risk of either type of adverse events. Of the 1.445 patients included in the ELDERLY-ACS 2 trial, more than two thirds (68%) had prior MI, diabetes or STEMI presentation, thus carrying a high thrombotic risk according to the ARC-HBR trade-off model [72]. These data show how HBR and high thrombotic risk coexist in a large number of elderly patients with ACS. 

## 5. Antiplatelet Strategies in Elderly ACS Patients 

In a recent review on antiplatelet therapy in ACS [73], we propose different DAPT strategies according to the presence or absence of HBR and high thrombotic risk. As discussed above, in elderly patients only two conditions are to be considered: (1) isolated HBR, and (2) HBR associated with high thrombotic risk.

**Figure 1:**
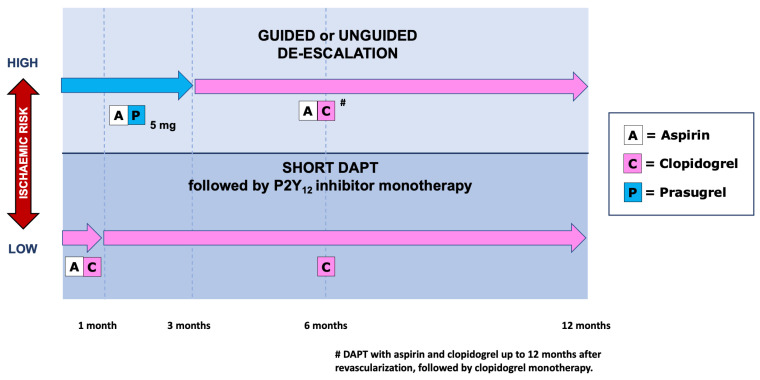
Algorithm for antithrombotic treatment strategies in elderly patients with acute coronary syndrome stratified by ischaemic risk. Abbreviations: DAPT, dual antiplatelet therapy.

For patients with isolated HBR, short DAPT is likely to be the best strategy (Figure 1). In the MASTER DAPT trial [74] that selectively randomized HBR patients (69% aged ≥75 years, 48% with ACS) to 1-month DAPT versus standard DAPT (median 157 days) followed by single antiplatelet agent (mostly clopidogrel in both groups), the abbreviated DAPT strategy was non-inferior to standard therapy for net adverse clinical events (NACE) and for ischemic events, but significantly reduced for major or clinically relevant non-major bleeding. This trial, however, also included patients taking anticoagulants (39%), for whom guidelines recommend an early DAPT cessation (1 week). The 1-month DAPT trial showed similar data [75]; that is, non-inferiority of short DAPT versus standard (6- to 12-month) DAPT followed by aspirin monotherapy for the 1-year composite of cardiovascular events or major bleeding in patients undergoing PCI for non-complex lesions [75]. However, in that trial a significant interaction was observed between treatment strategy and clinical presentation: ACS patients randomized to 1-month DAPT, contrary to stable ones, showed a numerical increase in cardiovascular events with no difference in bleeding as compared to standard-DAPT patients. These data caution against very short (1-month) DAPT periods followed by aspirin monotherapy in ACS patients [76]. 

Clopidogrel seems the most suitable P2Y12 inhibitor in older patients with HBR, due to a better safety profile than ticagrelor [53,77] and to an efficacy similar to ticagrelor [53,78] or low-dose prasugrel [38]. After DAPT cessation, clopidogrel may be preferred to aspirin as an antiplatelet monotherapy [79].

The higher risk of gastrointestinal discomfort or bleeding associated with aspirin is particularly evident in older patients [80]. This effect may result in a higher medication-discontinuation rate, a condition independently associated with increased mortality [81]. A higher adherence to clopidogrel than to aspirin was observed in the HOST EXAM trial, in which clopidogrel monotherapy was found to be superior to aspirin monotherapy as a chronic maintenance therapy among patients who had successfully completed the required duration of DAPT therapy after PCI [82]. Lower rates of both thrombotic and bleeding outcomes with clopidogrel as compared to aspirin were confirmed in an extended follow-up of over 5 years, after randomization [83]. Moreover, clopidogrel has an off-target anti-inflammatory action that may act as a modulator of the atherothrombotic risk [84,85]; this effect may be particularly beneficial in older patients, in whom frailty is frequently associated with a chronic low-grade inflammation (“inflammaging”), based on immunosenescence [18,86].

In patients with HBR associated with a high thrombotic risk (according to the variables included in the ARC-HBR trade-off model) [71] de-escalation appears as the most appropriate strategy. In a recent meta-analysis [87], de-escalation was superior to short DAPT for protecting against recurrent MI, and significantly reduced bleeding as compared to standard DAPT; a Bayesian meta-analysis showed that short DAPT ranked first in decreasing major bleeding, while de-escalation was first for NACE reduction, indicating that this strategy offers a balanced protection when both high thrombotic and high bleeding risks coexist [73]. Moreover, in a post hoc analysis of the Elderly ACS-2 trial, we found that low-dose prasugrel reduced ischemic events in the subacute (first month after index event) and chronic (from second month to 1 year) phases compared with clopidogrel, whereas bleeding complications were lower with clopidogrel in the late phase [50,88]. 

We believe that prasugrel low-dose rather than ticagrelor is the most suitable P2Y12 inhibitor to use in association with aspirin in the first 2–3 months after the index event in patients with HBR associated with a high thrombotic risk. A comparison between low-dose prasugrel versus standard-dose ticagrelor in elderly and low-weight ACS patients was performed in a sub-analysis of the ISAR-REACT 5 trial [89]. The results showed a numerical reduction in the primary-efficacy end point (12.7% of patients assigned to receive prasugrel and 14.6% of those assigned to receive ticagrelor; HR 0.82; 95% CI, 0.60 to 1.14). The difference was non-statistically significant, but P of interaction for comparison with the results observed in the group of younger and non-low weight patients (in whom there was a 35% significant reduction in the efficacy endpoint, favouring low-dose prasugrel) was also non-statistically significant. These data were associated with a non-significant decrease in major (BARC type 3 or 5) bleeding in the low-dose prasugrel group. Moreover, ticagrelor was found to increase bleeding complications as compared to clopidogrel in octogenarian patients included in a registry of NSTE-ACS patients [77].

After an initial period of 2 to 3 months, a switch from prasugrel low-dose to clopidogrel can be carried out. In the PRAGUE-18 trial, which tested prasugrel versus ticagrelor-based DAPT in patients (mean age 61.8 years) with MI, an economically motivated early switch to clopidogrel was not associated with an increased risk of ischemic events [90]. Therefore, in the light of these observations and of the fact that the thrombotic risk is high during the first months after the index event and wanes gradually over time [91], whereas the bleeding risk remains constant [92], it seems reasonable to propose a de-escalation strategy in older ACS patients with both high bleeding and high thrombotic risks, starting with DAPT including aspirin and low-dose prasugrel, then switching after 2–3 months to DAPT with aspirin and clopidogrel (Figure 1). After 12 months, clopidogrel monotherapy should be pursued.

## 6. Conclusions

The combination and duration of antiplatelet therapy in elderly patients with ACS is still a challenging issue, since most of these patients have both high bleeding and a high thrombotic risk. The evidence so far accumulated in the few studies involving this population favours a cautious approach, avoiding the use of powerful antiplatelet drugs such as full-dose prasugrel or ticagrelor. The suggestions expressed above and summarized in Figure 1 are mostly speculative, based on post hoc analyses from dedicated studies or from studies performed in general ACS populations. Randomized trials addressing the effects of therapeutic schemes based on the individual risk of elderly patients are needed, to clarify this issue. 

## Figures and Tables

**Table 1:** Randomized clinical studies comparing invasive versus conservative management of elderly patients admitted for ACS with or without persistent ST-segment elevation.

Study, Year of Publication, Ref.	Population	Number of Patients	Treatment Arms	Primary Endpoints	Main Results	Follow-up (Months)
**STEMI**						
TRIANA trial, 2011 [24]	Patients ≥75 years of age with STEMI presenting within 6 h of symptoms onset.	266	pPCI	All-cause mortality, re-infarction, or disabling stroke.	▪ Prematurely stopped, due to slow recruitment. ▪ No significant differences in the primary or secondary endpoint. ▪ Rate of recurrent ischaemia lower in pPCI-treated patients. ▪ No differences in major bleeding between the two groups. ▪ A pooled analysis with the two reperfusion trials showed an advantage for pPCI over fibrinolysis in reducing the primary endpoint at 30 days.	1 month
			Fibrinolysis			
Zwolle MI study group, 2002 [25]	STEMI patients of ≥75 years of age.	87	pPCI	Death, reinfarction or stroke at 30 days.	▪ pPCI resulted in lower rates of the primary composite endpoint, compared with fibrinolysis, at 30 days (RR: 4.3, 95% CI: 1.2 to 20.0; *p* = 0.01) and 1 year (RR: 5.2, 95% CI: 1.7 to 18.1; *p* = 0.001). ▪ No differences in the rate of noncerebral bleeding was detected.	1 year
			Fibrinolysis			
SENIOR PAMI, 2005 [26]	STEMI patients of ≥70 years of age.	483	pPCI	Death or disabling stroke at 30 days	No differences in the primary composite endpoint (11.3% vs. 13%, *p* = 0.57) or in-hospital major bleeding (5.6% vs. 6.2%, *p* = 0.79)	30 days
			Fibrinolysis			
**NSTEMI**						
TACTIS-TIMI 18, 2001 [29]	UA or NSTEMI patients (age ≥65 years in 43.5% of patients).	2220	Early invasive strategy (routine catheterization within 4 to 48 h and revascularization).	Death, nonfatal MI, and rehospitalization for ACS.	▪ All patients were treated with aspirin, heparin, and tirofiban. ▪ An early invasive strategy was associated with significantly lower rates of the primary composite endpoint (OR: 0.78; 95% CI: 0.62–0.97; *p* = 0.025). ▪ Protocol-defined bleeding occurred less frequently in patients randomized to a conservative strategy, albeit the rate of TIMI major bleeding did not differ between the groups.	6 months
			Conservative strategy (catheterization was performed only in case of recurrent ischemia or an abnormal stress test).			
FRISC II, 1999 [30]	NSTEMI patients (median age 66 years).	2457	Early invasive strategy (coronary angiography and, if appropriate, revascularisation, within 7 days from admission).	Death or MI.	▪ An early invasive strategy resulted in lower risks of the composite endpoint (RR 0.78; 95% CI 0.62–0.98, *p* = 0.031). ▪ An early invasive strategy also reduced angina and rehospitalization. No differences in major bleeding events were observed.	6 months
			Non-invasive conservative strategy.			
ICTUS, 2005 [30]	NSTEMI patients (age ≥65 years in 44.5% of patients).	1200	Early invasive strategy (coronary angiography within 24 to 48 h and revascularization).	Death or MI.	♦ Cumulative death or MI rates were 22.3% and 18.1%, respectively (HR: 1.29, 95% CI]: 1.00 to 1.66, *p* = 0.053). ♦ No difference was observed in mortality (HR: 1.13, 95% CI: 0.80 to 1.60, *p* = 0.49) or MI (HR: 1.24, 95% CI: 0.90 to 1.70, *p* = 0.20). ♦ Major bleeding occurred more frequently in patients randomized to an early invasive strategy (3.1%) compared with those who received a selective invasive strategy (1.7%).	5 years
			Selective invasive strategy (angiography and revascularization in case of refractory angina, hemodynamic or rhythmic instability, or clinically significant ischemia on the pre-discharge exercise test).			
RITA-3, 2005 [30]	Patients with NSTE-ACS (mean age 62 years).	1810	Early intervention	Two co-primary endpoints: Death, non-fatal MI, or refractory angina at 4 months Death or non-fatal MI at 1 year.	♦ An invasive strategy resulted in lower rates of the co-primary endpoint of death, MI or refractory angina at 4 months (RR 0.66, 95% CI 0.51–0.85, *p* = 0.001). ♦ The rate of death or MI at 1 year was comparable between the groups.	1 year
			Conservative strategy			
MOSCA	NSTEMI aged ≥70 years of age with at least two additional comorbidities.	106	Invasive strategy	All-cause mortality, reinfarction and readmission for cardiac cause.	♦ No differences in the primary endpoint between the groups. ♦ An invasive strategy resulted in lower rates of mortality and of mortality or ischemic events.	2.5 years
			Conservative strategy (coronary angiogram only if recurrent ischemia or heart failure).			
Elderly ACS trial, 2012 [13,31]	NSTEACS patients ≥75 years of age	313	Invasive strategy (coronary angiography within 72 h and revascularization if indicated).	Death, MI, disabling stroke, and repeat hospitalisation for cardiovascular causes or severe bleeding.	♦ No difference in the primary composite outcome (HR:0.80, 95% CI: 0.53–1.19, *p* = 0.26). ♦ An early invasive strategy resulted in lower rates of the primary outcome in patients with elevated troponin on admission. ♦ The rate of bleeding was low, and comparable among groups.	1 year
			Conservative strategy (coronary angiography if they demonstrated persistent myocardial ischemia, heart failure, or ventricular arrhythmias)			
After Eighty trial, 2016 [32]	UA or NSTEACS patients ≥80 years of age	457	Invasive strategy (including early coronary angiography with immediate assessment for PCI, CABG, and optimum medical treatment).	MI, need for urgent revascularisation, stroke, and death.	♦ Invasive strategy was significantly superior to conservative approach with respect to the primary endpoint (40.6% vs. 61.4%, HR = 0.53, 95% CI: 0.41–0.69, *p* = 0.001). ♦ No differences in the rate of major bleeding among the groups.	1.5 years
			Conservative strategy (optimum medical treatment alone).			
RINCAL trial, 2021 [33]	NSTEACS patients ≥80 years of age	251	Intervention-guided strategy plus OMT	All-cause mortality and non-fatal MI.	♦ No differences in the rate of the primary composite endpoint or major bleeding among groups.	1 year
			OMT alone			

Abbreviations: CABG, coronary artery bypass grafting; CI, confidence interval; HR, hazard ratio; MI, myocardial infarction; NSTE-ACS, non-ST-segment elevation acute coronary syndrome; NSTEMI, non-ST-segment elevation myocardial infarction; OMT, optimal medical therapy; pPCI, primary percutaneous coronary intervention; RR, relative risk; STEMI, ST-segment elevation myocardial infarction; TIMI, Thrombolysis in Myocardial Infarction; UA, unstable angina.

**Table 2:** Key contemporary randomized trials on DAPT with different P2Y_12_ inhibitors among elderly patients with ACS.

	Elderly ACS 2 Trial [49]	Triton-Timi 38 [42]	Plato [45]	Popular Age [53]
Year	2018	2007	2009	2020
Population	Elderly (>74 years of age) patients with ACS undergoing PCI.	ACS patients undergoing invasive management.	Sub-analysis of the PLATO trial in elderly (≥75 years) versus non-elderly (<75 years) patients.	Patients aged 70 years or older with NSTE-ACS.
Intervention(s)	Prasugrel 5mg + ASA (N = 2531)	ASA + prasugrel (N = 6813)	Ticagrelor 90 mg bid (N = 9333)	Clopidogrel 75 mg plus standard of care (N = 500)
Control	Clopidogrel 75 mg + ASA (N = 2514)	ASA + clopidogrel (N = 6795)	Clopidogrel 75 mg (N = 9291)	Ticagrelor 90 mg bid plus standard of care (N = 502)
Primary endpoint(s)	Death, MI, disabling stroke, or rehospitalization for CV causes or bleeding.	CV death, MI, stroke.	Death from vascular causes, MI, or stroke.	Net clinical benefit (all-cause death, MI, stroke and PLATO major or minor bleeding).
Safety endpoints	BARC 2, 3 or 5 bleeding.	Non-CABG-related TIMI major bleeding.	Trial-defined major bleeding.	PLATO major or minor bleeding.
Main results	▪ Enrollment interrupted prematurely, due to futility for efficacy. ▪ No differences between groups in the primary endpoint (HR: 1.007; 95% CI, 0.78–1.30; *p* = 0.955). ▪ Lower rates of definite/probable ST rates with prasugrel (OR: 0.36; 95% CI, 0.13–1.00; *p* = 0.06). ▪ Higher rates of BARC types 2 and greater with prasugrel (OR:1.52; 95% CI: 0.85–3.16; *p* = 0.18).	▪ DAPT with prasugrel 10 mg significantly increased major bleeding.	▪ The clinical benefit of ticagrelor over clopidogrel was not significantly different between patients aged ≥75 years of age (n = 2878) and those <75 years of age (n = 15,744) with respect to the primary composite endpoint or trial-defined major bleeding.	▪ Higher rates of drug discontinuation in the ticagrelor group. ▪ Clopidogrel resulted in significantly lower rates of the primary bleeding outcome (HR 0.71, 95% CI 0.54–0.94; *p* = 0.02 for superiority). ▪ Clopidogrel met non-inferiority for the co-primary net clinical benefit (absolute risk difference −4%, 95% CI −10.0–14; *p* = 0.03 for non-inferiority) compared with ticagrelor.
Follow-up	12 months	15 months	12 months	12 months

Abbreviations: ACS, acute coronary syndrome; ASA, aspirin; BARC, Bleeding Academic Research Consortium; CABG, coronary artery bypass grafting; CI, confidence interval; CV, cardiovascular; HR, hazard ratio; NSTE-ACS, non-ST-segment elevation acute coronary syndrome; OR, odds ratio; PCI, percutaneous coronary intervention; ST, stent thrombosis; TIMI, Thrombolysis in Myocardial Infarction.

## Data Availability

Not applicable.
